# HIV/AIDS in Russia: determinants of regional prevalence

**DOI:** 10.1186/1476-072X-6-22

**Published:** 2007-06-06

**Authors:** Dominique Moran, Jacob A Jordaan

**Affiliations:** 1 School of Geography, Earth and Environmental Sciences, University of Birmingham, Edgbaston, Birmingham, UK; 2 Department of Economics, Free University of Amsterdam, De Boelelaan 1105, 1081 HV Amsterdam, The Netherlands

## Abstract

**Background:**

The motivation for this paper is to inform the selection of future policy directions for tackling HIV/AIDS in Russia. The Russian Federation has more people living with HIV/AIDS than any other country in Europe, and nearly 70% of the known infections in Eastern Europe and Central Asia. The epidemic is particularly young, with 80% of those infected aged less than thirty, and no Russian region has escaped the detection of infections. However, measures to address the epidemic in Russia have been hampered by late recognition of the scale of the problem, poor data on HIV prevalence, potentially counterproductive narcotics legislation, and competing health priorities. An additional complication has been the relative lack of research into the spatial heterogeneity of the Russian HIV/AIDS epidemic, investigating the variety of prevalence rates in the constituent regions and questioning assumptions about the links between the epidemic and the circumstances of post-Soviet transformation. In the light of these recent developments, this paper presents research into the determinants of regional HIV prevalence levels in Russia.

**Results:**

Statistical empirical research on HIV and other infectious diseases has identified a variety of factors that influence the spread and development of these diseases. In our empirical analysis of determinants of HIV prevalence in Russia at the regional level, we identify factors that are statistically related to the level of HIV prevalence in Russian regions, and obtain some indication of the relative importance of these factors. We estimate an empirical model that includes factors which describe economic and socio-cultural characteristics.

**Conclusion:**

Our analysis statistically identifies four main factors that influence HIV prevalence in Russian regions. Given the different nature of the factors that we identify to be of importance, we conclude that successful HIV intervention policies will need to be multidisciplinary in nature. Finally, we stress that further research is needed to obtain a better understanding of the statistical relations that we have identified; our empirical findings can serve as an important guide in these future research efforts, as they indicate which processes play an important role in regional HIV/AIDS prevalence rates in contemporary Russia.

## Background

The Russian Federation has more people living with HIV/AIDS than any other country in Europe, and nearly 70% of the known infections in Eastern Europe and Central Asia. The epidemic is particularly young, with 80% of those infected aged less than thirty, and no Russian region has escaped the detection of infections [1]. The global significance of Russia's HIV/AIDS epidemic has been recognised for some time, and more recently awareness of the potential threat has become widespread in Russia, with a 2005 public poll reporting 84% of respondents' views that HIV/AIDS is a "big problem" [1], increased appreciation of the potential threat of HIV/AIDS to Russia's commercial interests [2], reporting of child abandonment by HIV-infected mothers [3], and the organisation of various events intended to raise awareness about the HIV/AIDS epidemic, such as Russian Fashion Week, and the recent East European and Central Asian AIDS Conference in Moscow.

**Figure 1:**
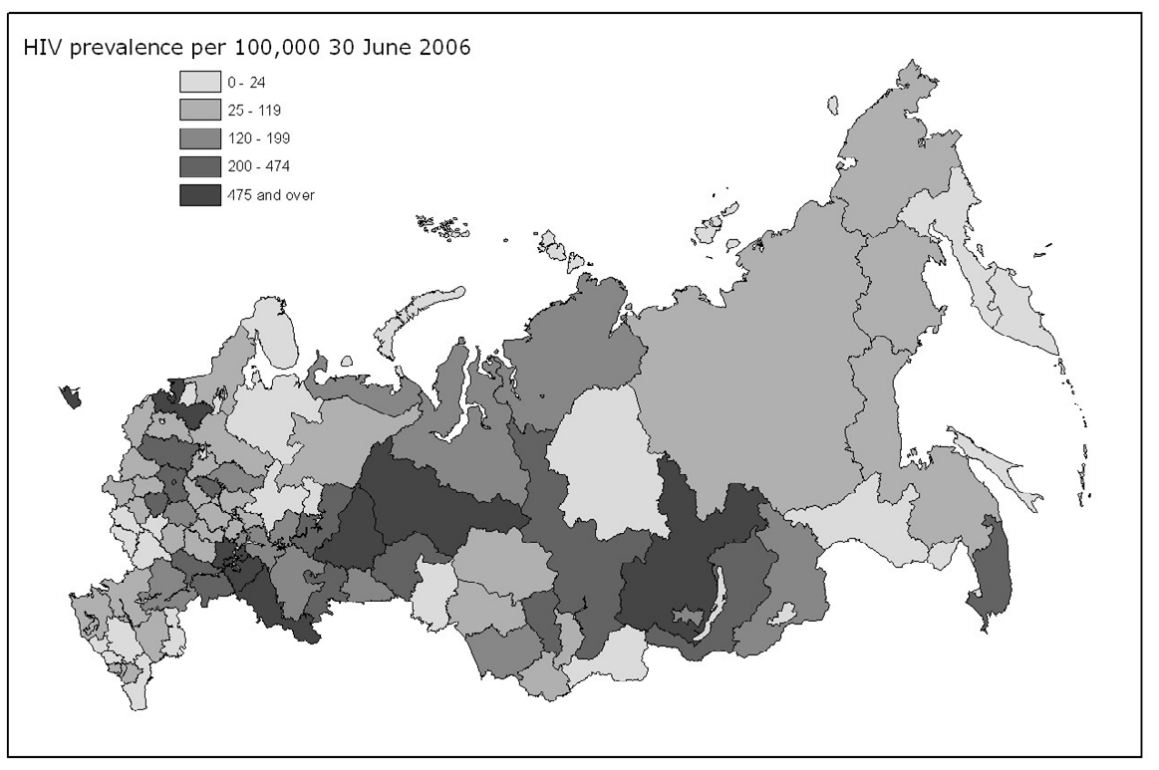
HIV prevalence per 100,000, 30 June 2006.

Political commitment to tackling HIV/AIDS within Russia is growing; over US$100 million has been allocated to tackling the epidemic in 2006, and public health is one of four priority projects for the Putin administration in 2006/7, with the Ministry of Health and Social Care set to prioritise HIV/AIDS. However, measures to address the epidemic in Russia have been hampered by late recognition of the scale of the problem, poor data on HIV prevalence, potentially counterproductive narcotics legislation, and competing health priorities. An additional complication has been the relative lack of research into the spatial heterogeneity of the Russian HIV/AIDS epidemic, investigating the variety of prevalence rates in the constituent regions and questioning assumptions about the links between the epidemic and the circumstances of post-Soviet transition. In the light of these recent developments, this paper presents research into the determinants of regional HIV prevalence levels in Russia. Figure 1 shows current regional variations in prevalence.

In a previous paper [4] this research gap was addressed through the presentation of results of exploratory analysis of the associations between HIV/AIDS prevalence and a range of socioeconomic measures reflecting transition, and some tentative explanations were offered for the distribution of prevalence rates. The paper found that the HIV epidemic is distinctly urban in distribution, and that there are strong and statistically significant relationships between HIV prevalence and indicators of regional economic development, domestic population movement and social 'dislocation', particularly amongst the age group of the population experiencing most infections. In the current paper, we extend this analysis, attempting to determine the relative importance of these independent variables, and to suggest potential policy implications of these findings.

There is a perceived association between HIV/AIDS and the circumstances of post-Soviet transition. Observers of the epidemic have commented, for example, that "social changes arising from political transition may have contributed to the spread of HIV" [5], and that the "upheavals of... economic and social dislocation, increased poverty, [and] new freedoms (including greater opportunities for geographic mobility, extramarital sex, prostitution, and drug use) [have] transformed the country into a far more conducive setting for the spread of HIV/AIDS" [6]. The HIV/AIDS epidemic in the CIS region has been observed by to be "predominantly... among urban, young, male injecting drug users and their sexual partners" [7]. Previous analysis [4] considered the various assumed characteristics of the epidemic, such as its urban character and connection to various aspects of transition, including long-term impoverishment, unemployment, loss of social cohesion, increased population mobility, and the regional expression of differential economic development during transition. Building on this research, and using the same datasets from Goskomstat Rossii and the Russian Federal AIDS Centre, this paper extends the investigation of the relationship between current HIV prevalence and past conditions. Measures of past rather than current conditions are included since there is a time lag of at least three months and possibly very much longer between infection with HIV and detection of this infection. Table 1 shows the dynamics of HIV infection in the Russian Federation since 1987.

**Table 1:** Number of reported HIV infections, 1987–2005

Year	Newly diagnosed HIV infections	**Cumulative total of infections**
1987 – 1994	887	**887**
1995	203	**1,090**
1996	1,513	**2,603**
1997	4,315	**6,918**
1998	3,971	**10,889**
1999	19,758	**30,647**
2000	59,261	**89,908**
2001	87,671	**177,579**
2002	49,923	**227,502**
2003	36,396	**263,898**
2004	32,147	**296,045**
**2005**	**37,287**	**333,332**

Source:

It is important to be clear about the limitations both of the data themselves, and the analysis which can be conducted. Official Russian HIV prevalence data are questionable in terms of their representation of the scale of the epidemic, and also vary in reliability through time. It is generally recognised that although official figures put the number of infections at over 375,000, actual figures are probably closer to 1.5 million. These prevalence data are generated from the HIV tests, and are therefore the prevalence of detections, rather than infections *per se*. Such data, which by necessity reflect detection of infection rather than 'actual' prevalence are in use elsewhere by researchers for the Russian Federation [8]. In terms of testing itself, there are concerns over the extent of targeting towards high-risk groups, and the precision of data reported to the centre [9]. However, while HIV prevalence data should be treated with caution because they do not represent HIV incidence, and because they depend heavily on patterns of HIV testing, which may vary across space and time, recent trends in HIV diagnoses in Russia are in general nevertheless unlikely to have resulted from changes in testing activities, because where such changes have been reported, the number of tests carried out has decreased rather than increased [10]; the decentralisation of HIV testing to the regions in 2002 has led to reductions in the numbers of tests being carried out [11]. For the purposes of this paper, it would be extremely unlikely that testing regimes would not differ between Russian regions, as they do between nations (although international comparisons are still considered relevant).

However, in the absence of a systematic review of testing procedure, regional data on numbers of tests carried out and amongst which groups of the population, it is impossible to determine the effect of this variation on the number of cases detected, and thus to control for this factor in the dataset. Therefore, these official HIV prevalence data are assumed to represent the same unspecified fraction of actual infections in all Russian regions. The potential weakness of the independent variables included in the analysis is also acknowledged; the reliability of Russian statistical data continues to be doubtful, socioeconomic coverage is not uniform, temporal coverage inconsistent and the 'shadow' economy is not accounted for [12].

In addition to the challenges posed by the data, the nature of HIV infection and the study of potential associations with explanatory variables means that caution must be exercised in interpreting results. HIV infection is caused by the transmission of the virus, through contact with the blood, or other bodily fluids of an infected person, or by mother to child during birth or via breastfeeding. Person-to-person transmission is predicated on risk behaviours (such as the sharing of injecting equipment, or unprotected sex) which do not of themselves *cause *HIV infection, but rather create circumstances in which infection is more likely if the virus is present. These risk behaviours may themselves be facilitated by certain contextual circumstances, including, arguably, the conditions of post-Soviet transition, and it is these associations which were tested by which will be the focus of the analysis presented here [4]. However, a further complication is that the effect of these contextual circumstances on risk behaviours is mediated by human agency. For example, the transmission of the HIV virus through commercial sex work (CSW) requires both a sex worker and a client. It is conceivable that the sex worker may have chosen this activity through poverty and lack of alternatives, whereas the client, perhaps a visiting business traveller, has sufficient disposable income to engage such services. In the same location, then, the co-existing circumstances of poverty and affluence converge to create the potential risk behaviour of commercial sex work – but the decision to engage in unprotected sex is down to choice, albeit again mediated by contextual circumstances such as power relations, economic considerations and the availability of protection. The analysis presented here cannot uncover the complex interplay of such influences – the estimated associations between the independent and the dependant variable cannot be envisaged as representing direct cause and effect relations – but what it can do is reveal the relative importance of a variety of risk factors (which approximate to descriptors of the contextual circumstances) in explaining the geographical distribution of HIV infections in the Russian Federation. Specifically, the contribution of this paper is to present and analyse findings of statistical analysis which isolates the effect of each independent variable, by controlling for the effect of other potential influences.

## Results

### Empirical model and data

Statistical empirical research on HIV and other infectious diseases has identified a variety of factors that influence the spread and development of these diseases [13-16]. In particular, these factors relate to economic and socio-cultural characteristics of countries or regions that affect the pattern and development of such diseases. Economic factors include life expectancy, income, gender inequality, labour mobility and education. Socio-cultural variables include religion, the ethnic composition of a population and the type of living environment. Other factors are the age of an epidemic, the types and availability of treatments of STDs and sexual practise, such as condom use.

However, a remark needs to be made here. While this analysis can determine the specific influence of an independent variable, it is important to consider that the effects of some of the variables are themselves multi-interpretational. For instance, the negative effect of the level of education on the prevalence of a contagious virus like HIV can be explained in several ways. A socio-cultural interpretation argues that an educated population is more likely to appreciate the risks of a contagious disease, and will be better able to understand the measures to prevent it. Alternatively, an economically-inspired argument would focus on the relation between income and education. Taking the positive effect of education on risk behaviour as given, the economic interpretation would argue that higher income countries have lower prevalence rates, due to their ability to provide for better education, although of course this is not always the case. This multi-interpretation indicates that a certain level of caution is required when interpreting the estimated effects of variables in quantitative research.

In our empirical analysis of determinants of HIV prevalence in Russia at the regional level, we want to look at two related issues. First, can we identify factors that are statistically related to the level of HIV prevalence in Russian regions, and second, can we obtain some indication of the relative importance of these factors? To address these questions, we estimate an empirical model that includes factors which describe economic and socio-cultural characteristics. This empirical model takes the following form:

Prevalence = β_0 _+ β_i _X_i _+ ε;

This model pictures the dependent variable prevalence as a linear function of a constant β_0_, a vector X containing a selection of independent or right hand side (RHS) variables and an error term, indicated by ε.

Prevalence is measured as the HIV prevalence rate in Russian regions for January 2005, per 100,000 inhabitants [4]. Ideally, we would like to estimate determinants of the regional prevalence rate for a number of years, which would allow us to see how the regional prevalence rates have developed through time. However, as these data are not available, we are confined in our analysis to perform a cross-sectional estimation of determinants of the prevalence rate.

The vector X contains a set of variables that we hypothesise to have a significant effect on the prevalence rate in the Russian regions. In our selection of RHS variables, we are guided by previous findings which present a set of individual correlations between the regional HIV prevalence level and variables capturing economic and socio-cultural circumstances [4]. However, we are somewhat restricted in our selection of variables for the present analysis, as many of the variables that could serve as RHS variable in our model are interrelated. For instance, several variables reflecting regional income and development levels are considerably correlated with each other, limiting the potential for their inclusion in the model, due to problems of multi-co-linearity, which would render significance statistics unreliable and hypothesis testing problematic [17]. In particular, for indicators of economic and social dislocation we had several potential proxy variables, such as income per capita, gross regional product (GRP), foreign direct investment (FDI) and various measures of the incidence of poverty, crime rates, drug crime statistics and so on. However, on initial analysis we found that many of these indicators are understandably very strongly correlated with each other and/or with the variable accounting for urbanisation.

Our solution to this problem is the following. Prior to our final selection of RHS variables for the empirical model, we experimented with transforming the variables, by taking natural logs and calculating variables per capita or per square kilometre. We then ran preliminary correlations and estimations of the model to look at the effects of the inclusion and exclusion of original and transformed variables. We have selected the variables which capture the effects of interest for the purposes of this paper, but which are not too closely interrelated. As a result of this preliminary analysis, we have selected five main RHS variables that we include in the empirical model.

The first two variables are largely economic. The variable GRP/Capita is the ratio of GRP divided by population for 2002, and it is used here as a proxy for income per capita. Observers of the HIV/AIDS epidemic in Russia have perceived a connection between the economic dislocation, stratification and regional inequality characteristic of the transition period [12,18-21] and the increase in prevalence of HIV [6,22]. Previous work has shown that while there is no constant pattern of association between HIV prevalence and indicators of economic development (such as unemployment, GRP, FDI), the strongest associations linked HIV prevalence and regional prosperity rather than impoverishment [4]. We expect that the estimated association between the prevalence rate and GRP/capita is negative, indicating that, *ceteris paribus*, HIV prevalence is lower in regions with higher income.

The second economic variable pertains to domestic population mobility, and represents the number of cars per 1,000 people in a region in 2003. This variable captures both an indication of economic development, and the level of mobility of a region's population, a descriptor of "new freedoms" [6]. In this statement, there is an element of individualism and independence of decision-making, reflected in geographic mobility. The choice of variable here was challenging, and among the possibilities was registration data, the recording of inter-regional migration which requires the re-registration of residence. This is the usual variable utilised to represent population movement [23]. However, our intentions here are to represent *opportunities *for population mobility in the widest sense, including local intra-regional mobility, and critically, more transient movement than can be captured by the registration data. Therefore, we tested the utility of data pertaining to the extent of metalled roads in a region, and also private car ownership, concluding that the latter was the more appropriate in terms of the statistical analysis. We expect the estimated effect of the mobility variable to be positive, indicating that higher mobility leads to a higher level of HIV prevalence.

The second set of variables touches on social and cultural influences. The first is the level of urbanisation of a region. This variable is the percentage share of the population in a region living in an urban area in 2003. The relationship between HIV and urbanisation in Russia has previously been noted, for example by the UNDP [7], and in previous research a strong and significant correlation between this variable and 2005 HIV prevalence has been demonstrated [4]. There are various reasons why an HIV/AIDS epidemic should be urban in distribution; risk behaviours may be assumed to be more prevalent in urban areas, and drugs more widely available. We again expect the estimated effect of the variable to be positive, in that as the percentage of a region's population resident in urban areas increases, so does HIV prevalence.

The second socio-cultural variable is the level of drug use in a region. In addition to an urban distribution, linkage between HIV and injecting drug use (IDU) is a major characteristic claimed for the Russian HIV/AIDS epidemic [24]. Although relationships between HIV prevalence and IDU have been identified in local studies [25-28], it is difficult to establish a statistical relationship due to the lack of data on levels of IDU. Very little data has been reported, and official figures are likely to be underestimates [29]. Given the lack of reliable regionally disaggregated data on IDU, a proxy would be the level of crime connected with illegal narcotics. However, in our initial analysis of potential RHS variables, this indicator was found to be highly correlated with the indicators of urbanisation and economic development. Therefore, we use the variable measuring crime committed by teenagers to proxy drug-related crime in a region. We expect to find a positive association between this variable and regional prevalence rate.

Teenager crime can also of course be an indicator of the observed process of social dislocation in transitional Russia. It is argued that social dislocation has been a factor increasing the likelihood of risk behaviours, but producing a statistical indication of the phenomenon is challenging. Crime and divorce rates have been used as indirect measures, assuming that high incidences indicate high levels of dislocation [4,30,31]. However, again, in initial analyses the various indicators of social dislocation were found to be strongly related to each other, with teenager crime showing the lowest levels of association. While this variable is used here primarily as a proxy for IDU, it may also be considered as an indicator of social dislocation.

We considered the inclusion of a variable to control for the variation in health care provision across the Russian regions, particularly the consideration of healthcare spending as a proxy for the level of HIV intervention activity. The selection of an appropriate variable was complicated by the fact that HIV prevention involves 'cheaper' aspects of the healthcare system, and is not easily approximated by the overall level of health spending [32]. There would be little logic, therefore, in assuming that HIV infection rates should fall as local overall health spending increases in the Russian context. An additional complication is the lack of data pertaining to healthcare spending itself; the best available proxy variable was the number of hospital beds in each region. This variable is problematic, as it is more related to the regional need for health care spending rather than spending itself [33]. In response to this, one could argue that there may be scale effects from health care provision, in the sense that regions with similar health care spending levels per capita may differ in the effectiveness of their regional health care systems and also in the extent to which these systems may create external effects. For instance, a larger health care system may provide quicker and better health information, which could affect regional prevalence rates. Therefore, we decided to use this imperfect health variable in the form of number of beds per region in 2003 and assess its effect in the empirical analysis.

Finally, cross-country empirical studies on the development of HIV include the age of the epidemic, as this can be related to the speed of development [15]. While it is probable that the HIV/AIDS epidemic is older in some Russian regions than in others, given the lack of regionally disaggregated data for HIV prevalence for the earliest years of the Russian HIV/AIDS epidemic, and also the unreliability of data collection, such analysis is not possible at present for the Russian regions.

### Empirical findings

To recapitulate, our empirical model is the following:

Prevalence = β_0 _+ β_1 _GRP/capita + β_2 _Mobility + β_3 _Urbanisation + β_4 _DrugCrime + β_5 _Healthcare + ε

The estimator is Ordinary Least Square. Table 2 presents the main empirical findings. It is important to note that we have performed the regressions using standardised variables. The RHS variables are measured in different ways, making it difficult to assess the relative strength of their estimated effects. The use of standardised variables controls for the difference in definitions, and as a result, the estimated coefficients of the RHS variables can be directly compared.

**Table 2:** Determinants of HIV prevalence in the Russian regions

Variables	Economic	Socio-cultural	Epidemio- logical	Full	Far East dummy	**Urban regions**
GRP/capita	-0.32 (2.45)***	--	--	-0.27 (2.28)**	-0.13 (1.68)*	-0.17 (1.89)*
Cars	0.46 (3.56)***	--	--	0.27 (1.92)**	0.30 (2.07)**	0.35 (2.30)**
Urbanisation	--	0.27 (2.52)***	--	0.25 (1.86)*	0.25 (1.98)**	0.39 (2.15)**
Crime	--	0.35 (3.17)***	--	0.33 (2.26)**	0.27 (2.04)**	0.26 (2.01)**
Number of beds	--	--	-0.35 (3.36)***	-0.17 (1.01)	--	--
State dummy	--	--	--	--	-0.65 (2.72)***	-0.64 (2.39)***
Constant	-0.03 (0.32)	-0.01 (0.02)	0.006 (0.06)	-0.06 (0.08)	-0.01 (0.02)	**0.07 (0.10)**
Adj R^2^	0.19	0.28	0.11	0.32	0.35	**0.36**
F	7.27 (0.000)	17.9 (0.000)	11.31 (0.000)	7.68 (0.000)	6.99 (0.000)	**6.51 (0.000)**
**N**	**78**	**87**	**86**	**78**	**78**	**73**

All variables are standardised. Estimations are heteroscedasticity robust. Absolute values of t statistic in parentheses. ***, ** and * indicate significance at the 1, 5 and 10% acceptance level.

Sources: HIV prevalence data compiled by authors from information supplied by the Russian Federal AIDS Centre, 2005 and socioeconomic data derived from Goskomstat Rossii, 2004, pp. 44–45, 72–73, 79–80, 112–114, 146–147, 169–170, 326–327, 328–329, 334–335, 347–348, 674–675, 925–926.

The first column with estimated effects is the restricted model that only includes economic variables. Both the economic variables carry significant coefficients, and the signs are in line with expectations. The negative sign of the coefficient of the GRP variable indicates that a region with a higher level of GRP per capita has a lower level of HIV prevalence, *ceteris paribus*. This suggests that regional prosperity [and perhaps also income] affects the risk behaviour of individuals, in the sense that people become less prone to engage in risky activity that may lead to the contraction of the HIV virus. The estimated effect of the cars variable is positive, suggesting that as domestic population mobility increases, so does HIV prevalence. Comparing the magnitude of the coefficients of the two variables indicates that the cars variable has a stronger effect on the prevalence rate than the income variable.

Next, we estimate the model focusing on the socio-cultural variables in the form of the level of regional urbanisation and the level of teenager crime as a proxy for drug crime. The findings show that both variables have a significant association with the prevalence rate. Again, both effects are similar to those expected. The level of urbanisation carries a significant positive coefficient, indicating that regions that are relatively more urbanised have a higher prevalence rate compared to more rural regions. This finding confirms the assertion that HIV/AIDS in Russia has an important urban dimension [7]. Next, the level of drug crime also has a positive significant association with HIV prevalence. This estimated effect supports the notion that regions with higher levels of drug use, and given the nature of the drug crime proxy, perhaps also those that experience a higher level of social dislocation, experience a relatively high prevalence rate.

The next column contains the results of a bivariate regression model to assess the effect of the healthcare variable. The results indicate a significant negative association between regional health care and prevalence rates, suggesting as expected that better health care leads to lower prevalence rates. Having said this, this finding must be interpreted with great caution, given the measurement and interpretational problems with this variable discussed in the previous section.

The results of estimating the full model, including all three types of RHS variables, are presented in the next column. Most have significant associations with the dependent variable, and the nature of the associations remains unaltered. GRP/capita still has a negative effect on regional HIV prevalence, whereas urbanisation, car ownership and the level of drug crime all have a positive effect. The only variable that becomes insignificant is the proxy for regional health care spending, meaning that this potentially questionable variable is in any case unimportant in the full model.

The full model produces estimated significant effects of the economic and socio-cultural variables that are in line with our expectations. To see the relative importance of these factors, we can compare the magnitude of the estimated coefficients. The mobility variable carries the largest coefficient. This suggests that, compared to the other three variables, mobility has the strongest effect on HIV prevalence rate. Next, urbanisation, drug crime and GRP/capita are approximately equally important in their association with IHIthe dependent variable.

Finally, we have estimated several alternative versions of the empirical model, to ensure that the estimated effects are robust. One issue we looked at is whether there are structural differences between multi-regional areas. To do this, we have added dummy variables to the empirical model, representing the seven federal districts. Most of the dummies do not have a significant association. Perhaps this is not too surprising, as the federal districts in Russia are not integrated units from an economic, social, or cultural perspective [34]. However, the dummy variable of the Far Eastern federal district does carry a significant coefficient. The inclusion of the Far East dummy into the model does not change the estimated coefficients of the other RHS variables, except for the variable representing regional prosperity, whose coefficient is halved as a result of the inclusion. The importance of this is that these results show that GRP/capita is less important in its effect on HIV prevalence compared to the other three RHS variables.

Second, we have re-estimated the empirical model for urbanised regions only. Running the estimation for all regions assumes that the level of urbanisation has a similar effect in regions that are highly urbanised and regions that are more rural in population distribution. Given the notion that HIV in Russia may be a particularly urban phenomenon, we estimate the empirical model for the most urbanised regions. For this, we define an urban region as a region in which at least 65% of the population lives in an urban area [4]. The findings are presented in the last column of Table 2.

The findings show that the nature of the associations between the RHS variables and the regional prevalence rate are unchanged. An important difference between the estimated regression model for urban regions and the previous findings is that the estimated coefficient of the urbanisation variable has increased considerably. This is important, as it indicates a change in relative importance of the RHS variables in the regions with highly urban population distributions. Considering all regions, the mobility variable is most important. In the most urbanised regions, the level of urbanisation has relatively the strongest association with the prevalence rate.

## Discussion and conclusion

The motivation for this paper is to support the selection of future policy directions for tackling HIV/AIDS in the Russian Federation. This is an area of considerable current concern both within Russia and internationally, with projects such as 'Globus' aiming to develop an effective national strategy to counter the HIV/AIDS epidemic, organising awareness-raising campaigns among vulnerable groups of the general population, and supporting preventative actions amongst those at highest risk of infection; activities which aim to intervene at the level of human response to contextual circumstances. Additionally, the Russian government has recently committed $109 m to a national health project to prevent, diagnose and treat HIV and viral hepatitis. The issue of HIV/AIDS has rocketed from obscurity to the top of the political agenda, with 2006 seeing the first conference on HIV/AIDS in Russia held in Moscow, and the intention to discuss means of tackling the problem at G8 in St Petersburg in the same year.

Political acknowledgement, and commitment to projects and funding are clearly timely and essential, but this paper additionally argues that macro-level analysis is important in targeting such interventions, in order that resources are most effectively deployed. By suggesting factors which may have contributed to the development of the HIV/AIDS epidemic in high prevalence regions, this paper indicates which factors must be addressed in successful policies aiming to combat regional HIV/AIDS prevalence rates. We have shown that HIV prevalence in Russia is strongly associated with the process of urbanisation, particularly in already highly-urbanised regions; domestic population mobility; drug use; and social dislocation, and negatively associated with GRP/capita, with the association between HIV and mobility the most significant. Therefore, our findings suggest that while regional political commitment, and appropriate infrastructure are essential in supporting interventions, the design and implementation of measures intended to prevent future infections should be informed by a consideration of the macro-level processes at work.

Considering our empirical findings with respect to recommending future policies to enhance and strengthen anti-HIV interventions in Russia, we would like to highlight the following three points.

First, our analysis statistically identifies four main factors that influence HIV prevalence in Russian regions. Importantly, these factors constitute elements that are part of contemporary processes of social and economic developments in the Russian Federation. Therefore, our finding that urbanisation, mobility, crime and income growth are important factors influencing HIV/AIDS prevalence rates strongly suggest that policy makers aiming to combat the HIV/AIDS epidemic can simply not afford to ignore these factors, given their presence and association with development processes in contemporary Russia.

Second, given the different nature of the factors that we have identified to be of importance, successful policies will need to be multidisciplinary in nature. For instance, policies addressing the positive relationship between the level of urbanisation and prevalence rates of HIV/AIDS will have to consist of a variety of measures originating from several disciplines, including urban planning, regional economic development and social and economic geography. Furthermore, in designing and implementing these policies, a central issue will be whether or to what extent such policies must be designed and implemented at the national, regional or sub-national level. Our analysis has focused on statistically identifying factors that future policies must address. Although we did not address the question of how these policies will have to be planned and carried out, this issue is an important one and cannot be avoided. It is clear that in recent years the willingness and commitment to fight the AIDS epidemic in Russia has improved substantially. To ensure that future increased efforts will have the maximum effect, multi-disciplinarity and the level of centralisation and decentralisation deserve substantial consideration.

Finally, we want to stress that further research is needed to obtain a better understanding of the statistical relations that we have identified. The variables that we have used in our analysis are measured at a high level of aggregation and are likely to represent a variety of processes at the regional level. For instance, it is likely that the positive relationship between urbanisation and HIV prevalence reflects the composite effect of a variety of factors that are associated with the general process of urbanisation. Furthermore, future (regional) policies will have to contain a certain measure of flexibility, as it is likely that regions will differ substantially in the nature and extent of underlying processes of our aggregate variables. In other words, our analysis only provides evidence of the effect of aggregate processes at the regional level; a more in-depth analysis of these factors is likely to show a substantial level of regional heterogeneity. Therefore, further research is needed at a more detailed level to obtain a better understanding of the effects of factors at the regional level as identified in our analysis, to identify actual levels of regional heterogeneity and ultimately to provide a more informed basis for future successful policy design and implementation. Notwithstanding this, our empirical findings can serve as an important guide in these future research efforts, as they indicate which processes play an important role in regional HIV/AIDS prevalence rates in contemporary Russia.

## Competing interests

The author(s) declare that they have no competing interests.

## Authors' contributions

DM conceived of the study and participated in its design, and constructed the map. JAJ designed and performed the statistical analysis. Both authors drafted the final manuscript. All authors read and approved the final manuscript.
